# Improved Proton Conductivity of Chitosan-Based Composite Proton Exchange Membrane Reinforced by Modified GO Inorganic Nanofillers

**DOI:** 10.3390/nano14141217

**Published:** 2024-07-17

**Authors:** Xinrui Guo, Zhongxin Zhang, Zhanyan Liu, Hui Huang, Chunlei Zhang, Huaxin Rao

**Affiliations:** 1 College of Chemistry and Materials Science, Jinan University, Guangzhou 510632, China; guoxinrui99@163.com (X.G.); 18961096289@163.com (Z.Z.); 17762482531@163.com (Z.L.); huangh0223@163.com (H.H.); 2 The First Affiliated Hospital of Jinan University, Guangzhou 510632, China; zclsir@126.com

**Keywords:** fuel cells, proton exchange membrane, chitosan, graphene oxide, composite membrane, inorganic nanofiller

## Abstract

Non-fluorinated chitosan-based proton exchange membranes (PEMs) have been attracting considerable interest due to their environmental friendliness and relatively low cost. However, low proton conductivity and poor physicochemical properties have limited their application in fuel cells. In this work, a reinforced nanofiller (sulfonated CS/GO, S-CS/GO) is accomplished, for the first time, via a facile amidation and sulfonation reaction. Novel chitosan-based composite PEMs are successfully constructed by the incorporation of the nanofiller into the chitosan matrix. Additionally, the effects of the type and amount of the nanofillers on physicochemical and electrochemical properties are further investigated. It is demonstrated that the chitosan-based composite PEMs incorporating an appropriate amount of the nanofillers (9 wt.%) exhibit good membrane-forming ability, physicochemical properties, improved proton conductivity, and low methanol permeability even under a high temperature and low humidity environment. When the incorporated amounts of S-CS/GO are 9 wt.%, the proton conductivity of the composite PEMs was up to 0.032 S/cm but methanol permeability was decreased to 1.42 × 10^−7^ cm^2^/s. Compared to a pristine CS membrane, the tensile strength of the composite membrane is improved by 98% and the methanol permeability is reduced by 51%.

## 1. Introduction

Proton exchange membranes (PEMs), which can selectively transport protons and cations, are considered the “heart” of the proton exchange membrane fuel cell (PEMFC) and can directly govern the performance of fuel cells (FCs) [1]. At present, commercially hydrated perfluorosulfonic polymer membranes (such as Nafion) have gained tremendous attention because of some superior properties including excellent proton conductivity, strongly acidic sulfonic acid groups, good thermal stability, and distinct phase separation. Although Nafion is the current market leader, some shortcomings such as high cost, high synthesis difficulty, high methanol permeability, and the loss of proton conductivity at temperatures exceeding 80 °C inhibit its use in large-scale FC applications [2,3]. Therefore, inexpensive, easily available, and low permeable aromatic polymers (such as polyimide, polysulfone) have become promising alternative PEMs instead of perfluorinated polymer membranes [4,5,6]. 

Natural polymeric membranes are nontoxic and inexpensive natural candidates for PEMFCs that have the potential to replace these synthetic PEMs [2]. At present, chitosan (CS), an economically viable polysaccharide-based biopolymer with promising potential, has received extensive interest as an alternative membrane material for FC applications, mainly due to its inherent low methanol permeability (about 4.0 × 10^−7^ cm^2^ s^−1^) and ease of chemical modifications [7,8]. Nevertheless, CS-based PEM also suffers from some significant drawbacks because of its natural structure (such as a high swelling nature). First, compared with Nafion and some non-fluorinated aromatic PEMs, CS-based PEM has lower proton conductivity (about 0.001 S cm^−1^ at room temperature), which has difficulty meeting the requirements of PEMs. The low proton conductivity has been attributed to its highly crystalline nature, which affects mechanical stability and proton mobility due to the partial protonation of weak alkaline groups in its main chain [2,9,10]. Second, during membrane preparation, the hydrophilic groups (e.g., –NH_2_ and –OH) of CS generate strong intramolecular and intermolecular hydrogen-bonding interactions, which drive CS chains to form numerous crystalline regions and to be stationary under electric field [11,12]. Finally, pristine chitosan may undergo a sharp transformation from a hydrophobic state to a swollen state as the matrix pH becomes acidic, leading to the protonation of amino groups of chitosan [13,14], and hence weakened mechanical stability and reduced proton conductivity. Therefore, there is an urgent demand for developing chitosan-based membranes with reduced methanol permeability, enhanced proton conductivity, and self-humidifying characteristics for PEMFC applications. 

To address the CS-related issues in PEMs, many modification methods, such as sulfonation (to increase water uptake), phosphorylation (to enhance proton conductivity), chemical cross-linking, embedding inorganic particles, and blending with other polymers, have been utilized to improve some performance of CS-based PEMs [7,15,16,17,18,19]. In particular, incorporating inorganic additives as a filler (such as functionalized graphene oxide, SiO_2_, carbon nanotubes) in a CS-based matrix can play a significant role in increasing proton conductivity and decreasing methanol permeability of CS-based PEMs [2]. In this regard, Abdollahi et al. [7] developed a CS nanocomposite membrane consisting of sulfonated graphene oxide (SGO) nanosheets of 0.5–10 wt.%. These authors reported a 454% increase in the proton conductivity when 5 wt.% SGO nanosheets were incorporated, compared to pristine CS. SGO provides a continuous and facile proton transport path to overcome the lack of proton conduction in the membranes. In another study, Wang et al. [20] fabricated a composite membrane consisting of a solvent-free carbon nanotube fluid in a chitosan matrix through an ion exchange method. The chitosan/carbon nanotube composite membrane was simultaneously reinforced by 180% and strengthened by 300% compared to a pristine CS membrane. Although some advancements have been made in CS-based PEMs in the past couple of years, some challenges and significant difficulties including the dispersion and compatibility of inorganic fillers with CS-based membranes need to be solved and developed to enhance their performance in FCs.

Based on the above considerations, we fabricated a novel reinforced composite PEM consisting of CS as the matrix and sulfonated CS/GO (S-CS/GO) as the inorganic nanofillers in order to improve the performance of CS-based PEMs. S-CS/GO is first synthesized through a noncovalent self-assembly method under electrostatic interactions between CS and GO aqueous solution. The microstructure and physicochemical properties of the membranes are investigated in detail. Additionally, the effects of the incorporated amounts of the inorganic nanofillers on the methanol permeability and proton conductivity of the composite PEMs will be further evaluated at different temperatures.

## 2. Materials and Methods

### 2.1. Materials

Chitosan (Mw = 1000 kDa, degree of deacetylation: 95%) was purchased from Aladdin. Graphene oxide (1~5 layers) was supplied from Suzhou Hengqiu Graphene Co. Ltd. (Suzhou, China). Acetic acid was purchased from Changzhou Hengguang Chemical Reagent Co. Ltd. (Guangzhou, China). Chlorosulfonic acid, sulfuric acid were obtained from Tianjin Damao Chemical Reagent Factory (Tianjin, China). Hydrogen peroxide was supplied by Guangzhou Chemical Reagent Factory (Guangzhou, China). All other analytical grade reagents and solvents were analytical reagent grade and used without further purification. Deionized water was used to prepare all samples.

### 2.2. Preparation of CS/GO

**Scheme 1:**
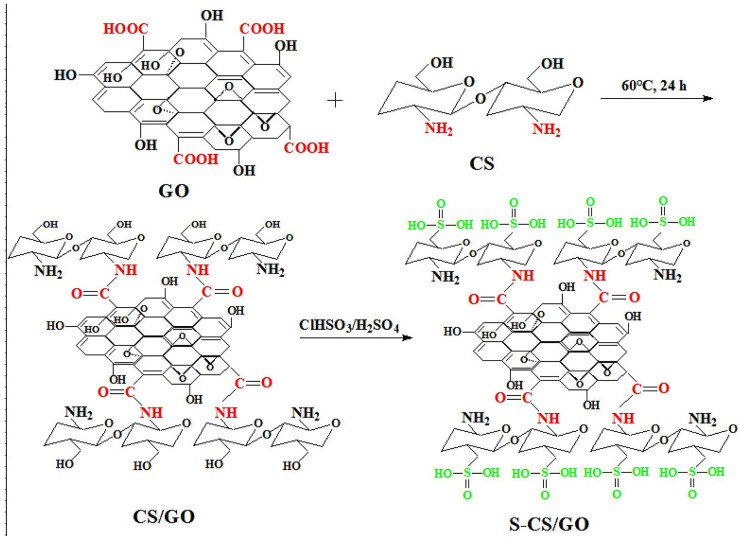
Synthesis process of CS/GO and S-CS/GO.

CS solution (1 wt.%) was prepared by dissolving CS in 1% (*v*/*v*) acetic acid solution. GO (0.02 g) was ultrasonically dispersed in 100 mL distilled water for 1 h to form an aqueous solution (0.2 mg/mL). After this, the CS solution was gradually added into the GO solution under a constant stirring, followed by an ultrasonic mixing at 60 °C for 24 h. The CS/GO product was collected by centrifugation and washed with copious deionized water until a neutral pH was achieved before it was freeze-dried to obtain a powder product. The CS/GO samples containing 1 wt.%, 2 wt.%, and 3 wt.% GO were correspondingly denoted as CS/GO-1, CS/GO-2, and CS/GO-3. The preparation process of CS/GO is pictorially illustrated in Scheme 1.

### 2.3. Preparation of S-CS/GO Nanofillers

Chlorosulfonic acid (20 mL) and 40 mL of sulfuric acid (40 mL) were mixed and cooled down to 4 °C to form a homogeneous solution. Then proper CS/GO was added to the solution and dissolved by stirring at room temperature for 1 h. The final product (sulfonated CS/GO, S-CS/GO) was precipitated by 200 mL of cold diethyl ether and then filtered. S-CS/GO was dissolved in deionized water and neutralized to pH 7 with 0.5 M NaHCO_3_ solution. The resulting solution was dialyzed in deionized water for 48 h and then freeze-dried to obtain the final product S-CS/GO, which was denoted as S-CS/GO-x, where x refers to the content of GO. The synthesis process of S-CS/GO is illustrated in Scheme 1.

### 2.4. Preparation of S-CS/GO@CS Composite PEMs

In order to investigate the effects of the type and the content of the nanofiller on properties of the CS-based PEMs, a series of S-CS/GO@CS composite PEMs and control CS membranes were prepared by fixing the type or the content of the nanofiller, respectively. The specific experimental preparation processes were as follows.

(1) S-CS/GO@CS composite PEMs by fixing the content of the enhanced nanofiller: 1 g CS was added to 30 mL of 3.33% (*v*/*v*) acetic acid solution and stirred at room temperature for 12 h to form a homogeneous solution. S-CS/GO-1 (67 mg) was added into 20 mL deionized water. After being ultrasonically dispersed for 1 h, the mixture was added into the CS solution over a period of 2 h and then stirred vigorously for 24 h to obtain a homogeneous membrane-forming solution. The S-CS/GO-1@15CS (denoted as SCG1/15CS) composite membrane was prepared according to the above membrane-forming conditions. S-CS/GO-2@15CS and S-CS/GO-3@15CS (denoted as SCG2/15CS and SCG3/15CS, respectively) composite membranes were also prepared by using the above way.

(2) S-CS/GO@CS composite PEMs by fixing the type of the nanofiller: The effects of the content of the nanofiller S-CS/GO on the performance of composite PEMs were further investigated. According to the above procedure, the S-CS/GO-1@10CS (denoted as SCG1/10CS) composite membranes were prepared by using 1 g CS and 0.1 g S-CS/GO-1. SCG1/15Cs and SCG1/7Cs composite membranes were prepared similarly. 

(3) Control CS membrane: 1 g of CS was dissolved in 50 mL of 1% (*v*/*v*) acetic acid solution and stirred vigorously at room temperature for 12 h to form a homogeneous solution. Then, the solution was cast in a mold to form a CS membrane. The control CS membrane was put into a vacuum oven at 60 °C for 24 h to remove excess solvent.

All dry composite membranes were incubated in 2 M NaOH solution for 30 min to remove residual acetic acid in the PEMs. The membranes are then washed with deionized water to neutralize. Finally, all the membranes with about 150 µm thickness were placed under vacuum oven at 25 °C for 24 h. 

### 2.5. Characterization

Transmission electron microscopy (TEM) was conducted to study the morphology of the samples using a JEOL JEM-2100F microscope (Japan Electronics Corporation, Tokyo, Japan) at an accelerating voltage of 200 kV.

X-ray diffraction (XRD) patterns of the composite PEMs were obtained using an X-ray diffractometer (MiniFlex, Nippon Rigaku Japan, Tokyo, Japan). The working conditions were Cu-Kα radiation source (λ = 0.154 nm), scanning angle 2θ in the range of 5–60°, and scanning speed of 2°/min.

The chemical structures of the samples were characterized by Fourier-transform infrared spectroscopy (FTIR) using NICOLET iS10 spectrometer from Thermo Scientific (Waltham, MA, USA) over the 4000−400 cm^−1^ range.

The thermal properties of the composite membranes were evaluated by thermogravimetric analysis (TGA) using a TGA2 analyzer from Netzsch, Selb, Germany. The temperature was scanned from 50 °C to 650 °C at a rate of 10 °C/min in a nitrogen atmosphere.

Zeta potential of the samples was measured by using zeta potential analyzer (Omni-type, Malvern, Arkansas).

An organic elemental analyzer (Flash 2000, Thermo Scientific, Selb, Germany) was used to determine the sulfonation yield of S-CS/GO.

### 2.6. Performance Measurement

#### 2.6.1. Measurement of Mechanical Properties

The mechanical properties of the composite membrane were measured using SHMADZU AG-1 of Kyoto City, Japan. The tensile rate was 2 mm/min, and every sample was tested three times under room temperature.

#### 2.6.2. Measurement of Oxidation Stability

The composite membranes were first immersed in 20 mL Fenton reagent (2 mg/L FeSO_4_ in 3% H_2_O_2_), and then placed into an oven at 80 °C to study their decomposition at different durations (40, 60, 80, 100, 120 min).

#### 2.6.3. Measurement of Water Uptake (WU) and Swelling Ratio (SR)

WU and SR were evaluated according to Equations (1) and (2), respectively, as previously reported by us [1,6].

(1) $$WU\left(\%\right)=\frac{W_{wet}-W_{dry}}{W_{dry}}\times 100\%$$

(2) $$SR\left(\%\right)=\frac{A_{wet}-A_{dry}}{A_{dry}}\times 100\%$$

where *W_dry_* and *A_dry_* are the weight and the surface area of the dry membranes, and *W_wet_* and *A_wet_* are the weight and the surface area of the wet membranes, respectively.

#### 2.6.4. Measurement of Ion Exchange Capacity (IEC), Proton Conductivity (σ), Selectivity and Methanol Permeability (P)

IEC, σ, and P were correspondingly evaluated using Equations (3)–(5), as described previously [1].

(3) $$IEC=\frac{C_{NaOH}\times V_{NaOH}}{W_{dry}}\times 100\%$$

where *V_NaOH_* is the volume of *NaOH* solution (as an indicator) consumed by titration (mL), and *C_NaOH_* is the concentration of *NaOH* solution (mol/L).

(4) $$\sigma =\frac{L}{R\times A}$$

where L (cm) is the distance between the two electrodes, R (Ω) is the resistance value measured by an AC impedance method, and A (cm^2^) is the area estimated by the thickness and width of the PEM samples.

(5) $$P=\frac{SV_{B}L}{AC_{A}}$$

where V_B_ denotes the volume of the solution in the B compartment (cm^3^), L is the thickness of the membrane sample (cm), A is the effective contact area between the membrane sample and the solution (cm^2^), and C_A_ is the molar concentration of the methanol solution in the A compartment (mol/L), S is the slope of the molar concentration–time curve of methanol solution in compartment B [1].

The selectivity (β, S s cm^−3^) of the composite membranes was evaluated by Equation (6), which is defined as follows,

(6) $$\beta =\frac{\sigma }{P}$$

## 3. Results

### 3.1. Structure and Performance of S-CS/GO Nanofillers

#### 3.1.1. Morphology Analysis

**Figure 1:**
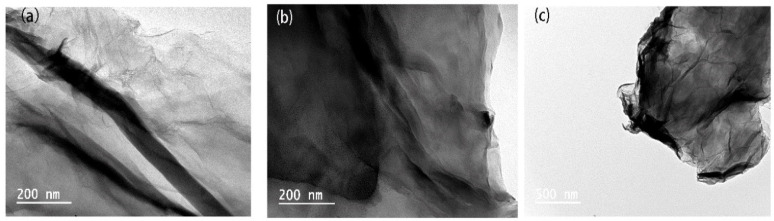
TEM images of GO (**a**), CS/GO-2 (**b**), and S-CS/GO-2 (**c**).

The surface morphologies of GO, CS/GO, and S-CS/GO were studied using TEM, and the results obtained are shown in Figure 1. It can be observed that GO exhibits a very thin and transparent monolayer structure and a folded surface morphology. However, the CS/GO and S-CS/GO surface morphology becomes rough and forms a multilayer structure by stacking from the original monolayer structure. It may be attributed to the electrostatic interaction between the amino groups in the CS structure and the carboxyl groups in the GO structure as well as the crosslinked entanglement between the GO layers.

#### 3.1.2. Chemical Structure Analysis

**Figure 2:**
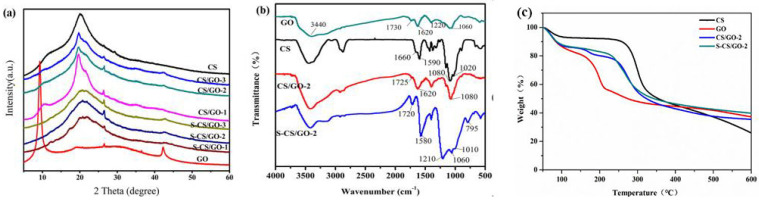
X-ray diffraction patterns (**a**), FTIR spectrum (**b**), and TGA curves of GO, CS/GO, and S-CS/GO (**c**).

The XRD patterns of GO, CS/GO, and S-CS/GO, shown in Figure 2a, were used to determine the quality of the nanofiller dispersion in CS-based PEMs. Pristine GO shows a sharp diffraction peak at 2*θ* = 11°, which is the diffraction peak of the GO (001) plane [21]. Pristine CS shows its characteristic broad peaks at 2*θ* = 14.9° and 20.3° due to its amorphous structure [22]. Both CS/GO and S-CS/GO have wide peaks with undefined CS morphology, but no characteristic peaks of GO appeared, indicating complete detachment of the GO lamellar structure and disappearance of the regular periodic structure [23]. When the GO content was changed, the chemical structures of CS in CS/GO and S-CS/GO remained almost unchanged. The wider peak shape and lower peak intensity of the S-CS/GO at 2*θ* = 20° compared to CS/GO and CS indicate that the sulfonation process can decrease the crystallinity of the S-CS/GO, which affects the mechanical stability and proton mobility. This is probably because the generated electrostatic interactions and hydrogen bonding result in a relatively ordered arrangement of CS chains between GO lamellae [24,25].

The FTIR spectra of GO, CS/GO-2, and S-CS/GO-2 are shown in Figure 2b. Compared with GO and CS, the intensity of the vibrational absorption peak of the C=O bond at 1730 cm^−1^ for CS/GO-2 and S-CS/GO-2 diminishes, the characteristic absorption peak of -NH_2_ on the CS side chain disappears, and the stretching vibrational peak of the amide bond appears at 1620 cm^−1^, indicating that GO/CS has been successfully synthesized. In addition, the intensity of the -OH absorption peak of S-CS/GO at 3340 cm^−1^ decreased significantly, indicating the formation of hydrogen bonding between CS and GO. The characteristic absorption peaks of the -SO_3_ group also appeared at 1210 cm^−1^, 1060 cm^−1^, and 795 cm^−1^, indicating that S-GO/CS has been successfully synthesized.

#### 3.1.3. Zeta Potential Analysis

The zeta potential of GO at pH = 7 was −27.17 mV, and the negative charge on its surface was attributed to the presence of a large number of oxygen-containing groups on its surface [26]. The Zeta potential of CS/GO increased to −22.22 mV due to the introduction of a nitrogen-containing amino group [27]. However, the zeta potential (−35.25 mV) of S-CS/GO is lower than those of GO and CS/GO, indicating that the surface of CS/GO has been successfully sulfonated.

#### 3.1.4. Thermal Stability Analysis

Figure 2c shows the TGA curves of GO, CS, CS/GO, and S-CS/GO. GO is thermally unstable and the major mass loss occurs close to 200 °C, which is attributed to the pyrolysis of labile oxygen-containing groups [28]. Compared to GO, GO/CS exhibited higher thermal stability due to the reduction of the carboxyl site on GO after binding to CS and the introduction of more polysaccharide structures [29]. The thermal performance of S-CS/GO was significantly enhanced due to the introduction of sulfonic acid groups at the hydroxyl site.

#### 3.1.5. Analysis of Organic Elements

The contents of carbon (C), hydrogen (H), nitrogen (N), and sulfur (S) elements in CS and S-CS/GO are shown in Table 1. It can be seen that the GO content in CS/GO gradually increased and the CS content gradually decreased as the GO content increased. In addition, due to the reduction of the CS macromolecular chain, the N element content in the S-CS/GO structure decreases, resulting in the reduction of sites where sulfonic acid groups were introduced and a decrease in the S element content. In conclusion, the sulfonation degree of S-CS/GO gradually decreased with an increase in GO content. Therefore, it can be speculated that the ratio of CS and GO should be controlled for S-CS/GO to obtain excellent-performance PEM-reinforced nanofillers.

### 3.2. Performance of the S-CS/GO@CS Composite PEMs

#### 3.2.1. Surface Morphology

**Figure 3:**
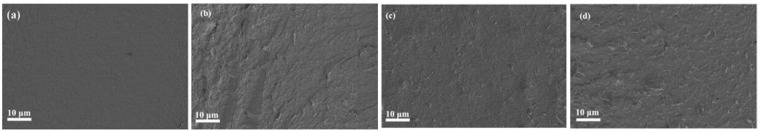
SEM images of cross-section for the CS and different CS-based PEMs ((**a**): CS; (**b**): CSG1/15CS; (**c**): SCG1/10CS; (**d**): SCG1/7CS).

Figure 3 shows the cross-sectional morphology of the CS membrane and different CS-based composite PEMs. The cross-section of the CS membrane is uniformly flat and smooth, but its poor mechanical properties make it fracture with uniform cracks in the cross-section (see Figure 3a). The internal structure of the composite membrane was changed by the incorporation of the S-CS/GO nanofiller. This is because the sulfonic acid groups and oxygen-containing groups enriched in the nanofiller structure can interact electrostatically with the polar groups (amino and hydroxyl groups, etc.) in the CS matrix with hydrogen bonding, thus forming a cross-linked network structure. Macroscopically, the composite membranes are uniform, rough, and dense. At the same time, the cross-linking between the two phases improves the mechanical properties of the composite membranes, and no cracks appear in any of their cross-sections.

#### 3.2.2. WU and SR

**Figure 4:**
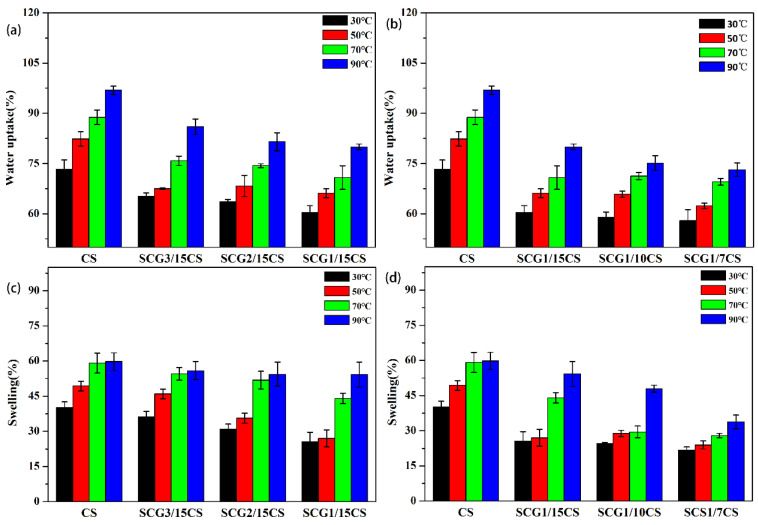
WU (**a**,**b**) and SR (**c**,**d**) of the CS and CS-based PEMs.

High WU would be beneficial for proton conduction, provided that it does not cause swelling problems of PEMs. The results WU of CS and CS-based composite membranes over a 30–90 °C range are shown in Figure 4a,b. It can be seen that the WU of all membranes increases with the increase in temperature. In addition, CS membranes have high WU due to the presence of many hydrophilic groups (such as -NH_2_, -OH), while all composite membranes have lower WU than CS. This is due to the restricted movement of the CS chains of the matrix arising from the hydrogen bonding and electrostatic interactions between the -SO_3_ groups on the surface of S-CS/GO and -NH_2_ [30]. At the same time, the free volume in the polymer matrix, which is mainly used for water storage is reduced, resulting in a decrease in the WU of the composite membrane [31].

At 30 °C, the WU of the SCG1/15CS, SCG2/15CS, and SCG3/15CS composite membranes was about 60.47%, 63.61%, and 65.30%, respectively. The WU of the composite membranes also increased with an increase in GO content. This is due to the presence of a large number of water-absorbing groups on the surface of GO, which confers good hygroscopicity [32], and the high content of GO in the nanofiller can absorb more water.

In addition, the WU of the SCG1/15CS, SCG2/10CS, and SCG3/7CS composite membranes was about 60.47%, 59.03%, and 57.99% at 30 °C, respectively. This is because the WU of the composite membranes gradually decreased with an increase in the S-CS/GO nanofiller content. This is because the free volume used for water storage in the composite membrane is further occupied with an increase in nanofiller. Meanwhile, the introduction of more -SO_3_ groups, with which more -NH_2_ on CS groups form hydrogen bonding and electrostatic interactions, further restricts the movement of CS group macromolecular chains, thus exhibiting lower WU.

Examples of the SR of CS membranes and different CS-based composite membranes over a 30–90 °C range are shown in Figure 4c,d. As seen in the figures, SRs of these membranes show the same trend as their WUs, most likely because a high WU will generally lead to a high SR [33]. CS membranes exhibit a higher SR due to their higher WU than composite membranes, while CS composite membranes show different degrees of decrease in their SR due to the different fillers added and their contents. This is due to the formation of hydrogen bonding and the electrostatic interaction between the incorporated nanofillers and the molecular chains of the matrix CS, making the membrane material more dense and less prone to swelling [34].

#### 3.2.3. Thermal Stability Performance

**Figure 5:**
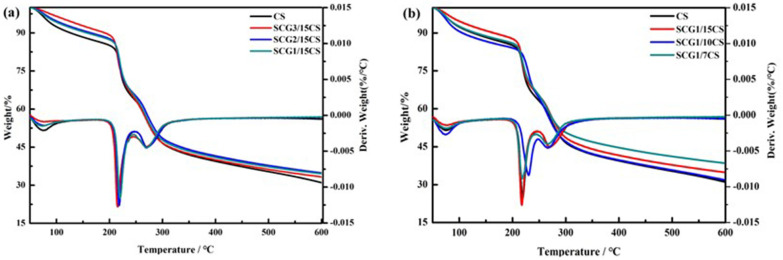
TG and DTG curves of SCG/15CS composite membranes with different nanofillers in the same amount (**a**) and different amounts of SCG1/CS composite membranes with the same nanofiller (**b**).

The thermal stability of PEMs obviously influences the performance of FCs and is characterized by TG to reveal their composition and changes. Specifically, the effects of the nanofiller type and content on the thermal stability of the composite membranes were investigated by TG, and the results are depicted in Figure 5. It can be seen that the thermal degradation behavior of the composite membranes is similar to that of pristine CS, indicating that the incorporation of the S-CS/GO nanofillers did not change the degradation mechanism of CS macromolecular chains. The thermal decomposition temperatures and amount of residual carbon at 600 °C for all membranes are listed in Table 2. It can be seen that the temperature at which the decomposition of the composite membranes starts is slightly lower when different S-CS/GO nanofillers are added. This is due to the addition of the S-CS/GO nanofiller, which disrupts the original crystalline structure in CS [30]. In addition, the starting decomposition temperature of the composite membrane gradually increased with the increase in the CS content of the nanofiller. This is due to the increase in the sulfonic acid groups on the side chains of S-CS/GO, which resulted in the strengthening of hydrogen bonding and the electrostatic interaction that formed between them and the CS matrix [12]. This led to an increase in the amount of residual carbon in the composite at 650 °C. Meanwhile, with an increase in nanofiller content, there was no change in the decomposition temperature at the beginning of the composite membrane, but there was a significant increase in the final carbon residue. It is shown that with an increase in nanofiller content, the stability of the CS macromolecular chain structure at high temperatures is enhanced to some extent.

#### 3.2.4. Mechanical Properties

The mechanical properties of PEM seriously affect the manufacturing conditions of FCs and their durability. Table 3 shows the tensile strength and elongation at the break of different CS-based composite membranes. From the table, it is clear that the incorporation of the nanofillers can significantly increase the tensile strength of the composite membranes.

As the GO content of the S-CS/GO reinforcing filler increases, the tensile strength of the composite membrane also increases. For example, the tensile strength of the S-CS/GO-3 composite membranes increased by 64% compared to pristine CS membranes. This is because GO has excellent mechanical properties and can be used as an excellent reinforcing agent for many polymers [35]. Meanwhile, the electrostatic and hydrogen-bonding interactions formed between the -NH_2_ and -OH on the CS side chains and the -SO_3_H groups on the S-CS/GO surface become a determining factor for the increased tensile strength. However, the elongation at the break of the composite membranes decreased with increasing GO content, indicating that the addition of small amounts of S-CS/GO nanofillers resulted in the membrane materials becoming stiffer and more brittle.

In addition, the tensile strength of the composite membranes increased and then decreased with the increase in S-CS/GO nanofiller content. The corresponding tensile strength of SCG-1/15 CS, SCGO-1/10 CS, and SCG-1/7 CS composite membranes was 26.97 MPa, 42.56 MPa, and 36.55 MPa, which was correspondingly 25%, 98%, and 70% higher than those of pristine CS membranes. This is because when more nanofillers are uniformly dispersed in the CS matrix, more physical cross-linking points can be generated through interfacial interactions, especially those of the amorphous phase, forming a cross-linked network. When subjected to external forces, interfacial stress transfer occurs between the two phases, dispersing the stress, while making the tensile strength of the composite membrane material further improved [14,36]. In contrast, when the excessive nanofiller is added, the tensile strength of the composite membrane is reduced due to the agglomeration of the nanofillers.

#### 3.2.5. Oxidation Stability

As H_2_O_2_ and degradation products -OH and -OOH with strong oxidative properties are produced during the operation of PEMFC and direct methanol fuel cells (DMFCs), oxidative degradation is considered the most important factor leading to the chemical and electrochemical degradation of the membrane [37]. Therefore, PEMs should have good oxidative stability to resist stronger oxidation conditions and thus improve the lifetime of FCs.

**Figure 6:**
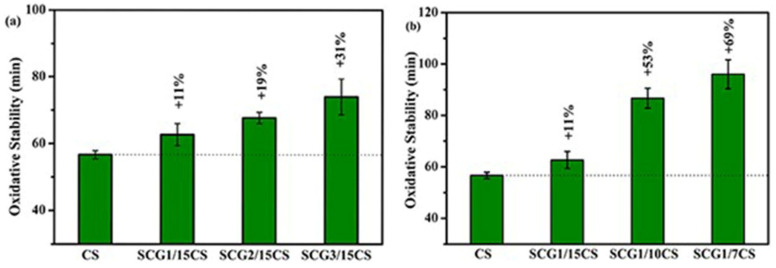
Oxidation stability of SCG/15CS composite membranes with different nanofillers in the same amount (**a**) and SCG-1/CS composite membranes with different amounts of the same incorporated nanofiller (**b**).

Figure 6 shows the time at which the decomposition of the composite membranes incorporated with different nanofillers and contents started to decompose in the Fenton reagent. It can be seen from the figure that both the incorporation and the increase in the content of the S-CS/GO filler can improve the oxidation resistance of the composite membranes. For example, after the incorporation of the SCG-3 nanofiller, the composite membrane SCG3/15CS started to decompose at 74 min, and its antioxidant stability was improved by 31% compared with pure CS. This is reasonably ascribed to the excellent antioxidant stability of the S-CS/GO nanofiller, and the functional groups on the surface of the nanofiller form strong hydrogen bonds and electrostatic interactions with the CS matrix, which to some extent weaken the oxidation of the CS matrix by -OH and -OOH reactive radicals in the Fenton reagent [38].

In addition, the increase in the content of the S-CS/GO nanofiller was able to improve the antioxidant properties of the composite membranes. After incorporation of 12.5 wt.% SCG-1 nanofiller, the antioxidant stability of the composite membrane SCG-1/7CS was increased by 69%. This may be due to the good compatibility between the two phases of the S-CS/GO nanofiller and CS matrix and the strong interactions generated to restrict the CS chain movement and delay the degradation of CS macromolecular chains.

#### 3.2.6. IEC

The IEC values of the composite membranes incorporated with different nanofillers and contents are listed in Table 4. It can be seen that the IEC of the composite membrane increases with the increase in the GO content of the nanofiller. This result is attributed to the sulfonic acid group contained in the nanofiller, which acts as a site for immobilizing protons and has a significant positive effect on the IEC of the composite membrane as its number increases [39].

In addition, the IEC of the composite membranes increases and then decreases with the increase in the content of the S-CS/GO-1 nanofiller incorporated. When the content of S-CS/GO-1 is added at 12.5%, the IEC of the composite membrane decreases to 0.64 mmol/g. This is because with the addition of excess S-CS/GO-1, they undergo a slight agglomeration in the matrix and GO tends to re-stack, thus inactivating some sulfonic acid groups between the nanosheet layers [39], thus exhibiting lower IEC values.

#### 3.2.7. *σ* and Conduction Mechanism

**Figure 7:**
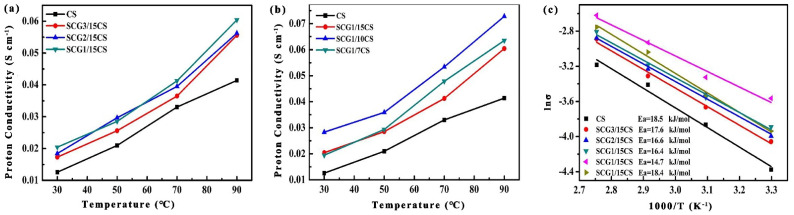
Proton conductivity of CS-based composite membranes with different nanofillers (**a**) and different amounts (**b**) at 100% RH and at different temperatures; Arrhenius plots of the composite membranes (**c**).

*σ* is a crucial parameter for evaluating the performance of FCs. Figure 7 shows *σ* of the composite membranes incorporated into different nanofillers and contents at different temperatures (100% RH humidity). From Figure 7a, it can be seen that *σ* of the composite membrane gradually increases with the increase in the CS ratio and sulfonic acid group in the S-CS/GO nanofiller. This is because, in the SCG/15CS composite membrane, the nanofiller is better dispersed in the CS matrix, which leads to a decrease in the crystallinity of the CS matrix and an increase in the amorphous phase. In addition, *σ* in the membrane occurs mainly in the amorphous phase [40].

Also, electrostatic interaction and hydrogen bonding between the CS matrix and the nanofiller help to promote the formation of continuous proton transport channels in the membrane, achieving rapid proton conduction through the Grotthuss mechanism [41]. Notably, the composite membranes have lower WU compared to the CS membranes, indicating fewer proton carriers and a decreased chance of protons crossing the membrane through the action of the Grotthuss mechanism. However, the introduction of a large number of sulfonic acid groups on the surface of the packed S-CS/GO increases its IEC and subsequently increases the sites for proton hopping. Therefore, the Grotthuss mechanism is considered to be the main reason for the enhanced *σ* of the composite membranes [7]. At the same time, *σ* depends not only on the effective conduction of protons, but also on the proton concentration. The increase in proton concentration due to the decrease in the water dilution effect is also one of the reasons for the increase in *σ* [39].

As can be seen in Figure 7b, the *σ* of the composite membrane increases gradually with an increase in nanofiller content and test temperature. Protons can pass through the membrane material quickly because the introduced hydrophilic sulfonic acid group ion clusters form well-connected channels in the membrane. However, when the nanofiller content is 12.5%, the aggregation of S-CS/GO in the CS matrix makes the proton conduction path convoluted and hinders the conduction of protons along the sulfonic-acid-based ion clusters, thereby reducing *σ* of the composite membrane to some extent. Therefore, there is an optimum amount of nanofillers for incorporation, which leads to a maximum *σ* of the composite PEMs.

The proton conduction mechanism is the key to the design and preparation of highly conductive PEMs, which can be reflected by the proton conduction activation energy (*E*a), the minimum energy required for a proton to pass through the membrane material. *E*a of a composite membrane was evaluated from an Arrhenius plot shown in Figure 7c. All those that exhibited a Grotthuss mechanism in their proton permeation behavior have *E*a in the range of 14.3~39.8 kJ/mol [42]. *E*a values of all composite membranes are in the range of 14.7~18.5 kJ/mol, which is smaller than that of CS membranes. This indicates that the proton conduction in all membranes is mainly controlled by the Grotthuss mechanism. As the sulfonic acid groups on the packing surface and the side chain amino groups of the CS matrix can form acid–base pairs, which provide new proton jumping sites and establish the pathway for effective proton transfer, the rapid transfer of low energy potential barrier protons is realized, thus improving the proton conduction performance. At the same time, with an increase in sulfonic acid groups in the nanofillers, more acid–base pairs sites for proton hopping are more easily formed in the composite membranes, leading to more Grotthuss proton migration, thus exhibiting a gradual decrease in *E*a [43]. However, when the excessive nanofillers of the composite membranes are incorporated, the inhomogeneous dispersion hinders the proton migration channel, while making *E*a appear to rise. The above phenomenon corresponded with the improved proton conductivity of the composite PEMs.

#### 3.2.8. Methanol Permeability and Selectivity

Methanol permeability is an important parameter that determines some performance of PEM in fuel cells, especially in DMFC. Excessive methanol permeating the membrane will yield a lower open circuit voltage and catalyst poisoning occurs, which will reduce FC performance. Therefore, proton exchange membrane materials with the lowest possible methanol permeability are often expected.

The methanol permeability of the composite PEMs incorporated with different nanofillers and contents are listed in Table 4. The methanol permeability of the pure CS membrane was 3.38 × 10^−7^ cm^2^/s, which was much lower than that of Nafion 117 (2.91 × 10^−6^ cm^2^/s). The methanol permeation of the composite membranes decreased with an increase in incorporated GO content in the S-CS/GO nanofillers and an increase in S-CS/GO content. It could be the reason that the incorporation of the nanofiller particles impeded the hydrophilic channels and decreased the migration of methanol in the composite membranes [41]. With the increase in sulfonic acid groups and oxygen-containing groups in the nanofiller, the hydrogen bonding and electrostatic interactions between the nanofiller and CS matrix were enhanced, narrowing the methanol diffusion channels. 

The selectivity values of the composite PEMs incorporated with different nanofillers and contents are also listed in Table 4. It can be seen that the incorporated S-CS/GO nanofillers can improve the selectivity of the composite PEMs. For example, the selectivity value of the SCG-1/10CS composite membrane is about five times greater than that of the pure CS membrane.

## 4. Conclusions

In summary, a novel CS-based composite PEM was successfully prepared by incorporating a reinforced S-CS/GO nanofiller into a CS matrix to overcome some defects of non-fluorinated CS-based PEMs. The results showed that the type and amount of the nanofillers had an obvious effect on the physicochemical and electrochemical properties of the composite PEMs. For example, when the incorporated amounts of S-CS/GO are 9 wt.%, the proton conductivity of the composite PEMs was up to 0.032 S/cm but methanol permeability was decreased to 1.42 × 10^−7^ cm^2^/s. Compared to a pristine CS membrane, the tensile strength of the composite membrane is improved by 98% and the methanol permeability is reduced by 51%. At the same time, the composite PEMs incorporating an appropriate amount of the nanofillers exhibit good membrane-forming ability, physicochemical properties, improved proton conductivity, and reduced methanol permeability even under a high temperature and low humidity environment. The improvement in comprehensive properties in the CS-based composite PEM can be attributed to the generated hydrogen bonding and electrostatic interaction between hydrophilic -SO_3_- groups in the nanofiller and -NH_2_ groups in the CS matrix. These results indicate that incorporating the reinforced S-CS/GO nanofiller into the CS matrix can generate a more accessible pathway for proton migration through the composite membrane and is efficient in designing novel CS-based PEMs for fuel cell applications.

## Figures and Tables

**Table 1:** Element content in CS and S-CS/GO with different structures.

Samples	GO and CS Mass Ratio	GO Content (%)	CS Content (%)	N Content (%)	C Content (%)	S Content (%)	H Content (%)
CS		0	100.00	7.57	40.39	0	7.15
S-CS/GO-1	1:15	6.25	93.75	1.97	46.36	3.81	3.48
S-CS/GO-2	1:10	9.09	90.91	1.55	49.43	3.45	3.26
S-CS/GO-3	1:5	16.70	83.30	1.32	48.90	3.29	2.90

**Table 2:** Initial decomposition temperatures and final residual carbon content at 600 °C of the different composite membranes.

Membrane Samples	SCG/CS (wt/wt)	Initial Decomposition Temperature (℃)	Final Residual Carbon Content (%)
CS		213.35	31.03
SCG-3/15CS	3/15	209.57	33.29
SCG-2/15CS	2/15	211.84	34.85
SCG-1/15CS	1/15	212.91	34.54
SCG-1/15CS	1/15	212.97	34.54
SCG-1/10CS	1/10	217.47	31.80
SCG-1/7CS	1/7	210.92	38.50

**Table 3:** Mechanical properties of different CS-based composite membranes.

Membrane Samples	SCG/CS (wt/wt)	Tensile Strength (MPa)	Elongation (%)
CS		21.5 ± 2.2	17.4 ± 2.5
SCG-1/15CS	1/15	23.8 ± 1.5	12.8 ± 1.7
SCG-2/15CS	1/15	26.9 ± 2.8	8.9 ± 1.5
SCG-3/15CS	2/15	35.3 ± 3.7	9.1 ± 2.5
SCG-1/10CS	1/10	42.6 ± 3.3	17.8 ± 1.4
SCG-1/7CS	1/7	36.6 ± 2.4	14.5 ± 2.8

**Table 4:** IEC, methanol permeability, and selectivity of the CS-based composite membranes.

Membrane Samples	IEC (mmol/g)	Methanol Permeability (*p*, × 10^−7^ cm^2^/s)	Selectivity (SP, ×10^4^ S·s·cm^−3^)
CS	0.38 ± 0.02	3.38 ± 0.58	3.72 ± 0.16
SCG-3/15CS	0.44 ± 0.05	2.33 ± 0.06	7.42 ± 0.28
SCG-2/15CS	0.54 ± 0.06	2.09 ± 0.26	8.82 ± 0.34
SCG-1/15CS	0.61 ± 0.03	1.86 ± 0.23	10.99 ± 0.51
SCG-1/10CS	0.74 ± 0.05	1.66 ± 0.36	17.01 ± 0.68
SCG-1/7CS	0.64 ± 0.04	1.19 ± 0.17	16.39 ± 0.71

## Data Availability

The original contributions presented in the study are included in the article, further inquiries can be directed to the corresponding author.
